# Advanced Pedestrian State Sensing Method for Automated Patrol Vehicle Based on Multi-Sensor Fusion

**DOI:** 10.3390/s22134807

**Published:** 2022-06-25

**Authors:** Pangwei Wang, Cheng Liu, Yunfeng Wang, Hongsheng Yu

**Affiliations:** 1 Beijing Key Lab of Urban Intelligent Traffic Control Technology, North China University of Technology, Beijing 100144, China; liucheng1130@126.com (C.L.); wuyu13522777537@126.com (Y.W.); yuhongsheng110@126.com (H.Y.); 2 Key Laboratory of Operation Safety Technology on Transport Vehicles, Research Institute of Highway, Ministry of Transport, Beijing 100088, China

**Keywords:** advanced sensing technology, multi-sensor fusion, crowd density estimation, movable temperature detection, automated patrol vehicle

## Abstract

At present, the COVID-19 pandemic still presents with outbreaks occasionally, and pedestrians in public areas are at risk of being infected by the viruses. In order to reduce the risk of cross-infection, an advanced pedestrian state sensing method for automated patrol vehicles based on multi-sensor fusion is proposed to sense pedestrian state. Firstly, the pedestrian data output by the Euclidean clustering algorithm and the YOLO V4 network are obtained, and a decision-level fusion method is adopted to improve the accuracy of pedestrian detection. Then, combined with the pedestrian detection results, we calculate the crowd density distribution based on multi-layer fusion and estimate the crowd density in the scenario according to the density distribution. In addition, once the crowd aggregates, the body temperature of the aggregated crowd is detected by a thermal infrared camera. Finally, based on the proposed method, an experiment with an automated patrol vehicle is designed to verify the accuracy and feasibility. The experimental results have shown that the mean accuracy of pedestrian detection is increased by 17.1% compared with using a single sensor. The area of crowd aggregation is divided, and the mean error of the crowd density estimation is 3.74%. The maximum error between the body temperature detection results and thermometer measurement results is less than 0.8°, and the abnormal temperature targets can be determined in the scenario, which can provide an efficient advanced pedestrian state sensing technique for the prevention and control area of an epidemic.

## 1. Introduction

At present, in order to curb the COVID-19 pandemic, governments have divided the current areas into disease control areas and normal areas according to the different status of the epidemic. The source of infection, which may break out in the disease control area, has an important impact on the daily life of the general public. The crowd aggregation and contact temperature measurement method based on hand-held thermometers increases the risk of cross-infection. Therefore, how to reduce the risk of infection for medical practitioners and pedestrians in disease control areas and ensure the effective implementation of epidemic prevention measures have become important issues. In order to solve the above problems, it is necessary to detect crowd aggregation and body temperature rapidly based on the advanced sensing technology. Due to the improvement in sensor accuracy and detection algorithms in recent years, sensing technology and application technology are developing rapidly. A connected and automated vehicle (CAV), which is equipped with sensors, such as a camera, radar and LiDAR, has been widely used in short-distance logistics distribution and security patrols to improve transportation efficiency and driving safety [1]. As one of the CAVs, the automated patrol vehicle can be used as a new solution for epidemic prevention and control. Therefore, how to improve the accuracy of sensing and apply high-precision sensing technology to the automated patrol vehicle have become key research directions.

However, there are still some problems in the above directions, which can be classified in the following conditions: (1) pedestrians in public areas, such as in parks, museums and shopping malls, do not have a fixed route, and the difference in crowd density distribution in adjacent areas is tiny, which makes it difficult to detect dense areas effectively; (2) there are technique and evaluation difficulties in the combination of high-precision crowd density estimation and body temperature detection. In order to solve the above problems, an advanced pedestrian state sensing method for automated patrol vehicles based on multi-sensor fusion is proposed. Considering the current pandemic situation and the limitations of the experimental platform, a large and enclosed area with less traffic flow is selected as the work scenario of the automated patrol vehicle. Firstly, through fusing the data of the camera and LiDAR, the multi-dimension data of pedestrians are obtained as the basis of density estimation and face detection. Secondly, the centroids of the clustered pedestrian objects are calculated as the cluster center point. Combined with the positional relationship between pedestrians, crowd targets are detected based on the foreground image. Then, the whole detection area is divided into multiple layers by various scales, and each sub-area of each layer is constructed as the basic unit of the multi-layer fusion method. Through weighted mean theory, the crowd density in the scenario is estimated based on the crowd density distribution matrix. Finally, because of the precision limitation of the sensor, the automated patrol vehicle needs to maintain a fixed distance from crowd targets, and a thermal infrared camera is used to detect the temperature, which can reduce the risk of cross-infection.

## 2. Related Works

The advanced sensing technologies lead to the rapid development of CAV, providing a platform foundation for mobile detection. Meanwhile, the gradual development of deep learning [2], pedestrian detection, crowd density estimation and pedestrian body temperature detection techniques have been hot topics.

Pedestrian detection is one of the important parts of automated vehicle sensing systems and has been researched for many years. It is mainly achieved through the neural network [3,4,5] and open-source computer vision library (OpenCV) [6]. Among them, the convolutional neural network (CNN) proposed by Lecun [7] was representative. Local pixels of the image were perceived by the network and combined as global features in higher layers. This meant that the pooling layer would pay more attention to local features and ignore the overall target. Multi-sensor data fusion methods [8,9,10] have been paid more and more attention in recent years. Multi-sensor data fusion is a method to combine data with multi-level and multi-spatial information and output a consistent interpretation of the targets. There are three levels of fusion, including: (1) data-level fusion; (2) feature-level fusion and (3) decision-level fusion. In order to reduce the false positive (FP) rate of pedestrians in a complex environment, Han et al. [11] integrated image data and point cloud data deeply through decision-level fusion to improve detection accuracy. Combining the object detection algorithm of you only look once (YOLO) and a light field camera, Zhang et al. [12] proposed an indoor obstacle detection algorithm. This algorithm was suitable for detection in the indoor environment but had not been tested in outdoor environments. In order to detect heavily occluded pedestrians, Seong et al. [13] proposed a fusion algorithm of LiDAR and radar, which avoided the strict requirements regarding the conditions of lighting for the camera, and it was suitable for indoor and outdoor environments.

Crowd density estimation has been paid more and more attention due to the requirements of abnormal event sources localization and the policy under the pandemic situation in recent years. With the advancement in sensor techniques, the current mainstream crowd density estimation is achieved through neural networks [14,15,16,17,18]. Huang et al. [19] proposed a multi-scale fusion conditional generative adversarial network based on a bidirectional fusion module. The network could solve the problem of scale variation effectively. In order to reduce crowd counting errors caused by large viewing angle changes and severe occlusions, Sajid et al. [20] proposed an end-to-end crowd counting network based on patch rescaling module (PRM) and multi-resolution fusion. In order to make deep features recognizable by CNN, Nguyen et al. [21] fused depth information into a density estimation algorithm. Through a multi-task learning method, the scale variation problem was solved and crowd counting was achieved accurately. Through applying an edge computing method, Wang et al. [22] achieved crowd density estimation efficiently to solve the problem of high network latency caused by the deployment of the density estimation platform in the server; Zhang et al. [23] proposed the adaptive multi-scale context aggregation network (MSCANet) to obtain the full-scale information of the crowd. After the information of different scales was extracted by the network, this information was fused by the network adaptively, which was suitable for crowd counting.

With the gradual development of automated vehicle sensing techniques and body temperature detection techniques, body temperature detection is gradually being applied in the automated vehicle [24,25]. At present, body temperature detection is mainly achieved by the method of thermal imaging [26,27]. With the continuous development of image processing techniques, thermal imaging technology combined with image recognition has become the mainstream method. Based on image processing, Muhammad et al. [28] proposed a pedestrian body temperature detection method to fuse infrared and visible images. The target and the background could be distinguished and the infection problem that might be caused by the gun-type thermometer was avoided by this method. Considering the difficulty of detecting body temperature without distance limitation, this method could measure temperature accurately by strictly fixing the temperature measurement distance and controlling environmental variables. Based on the micro control system 51 series (MCS 51) equipped with infrared temperature measurement function (GY-MLX90614 module), Zhu et al. [29] designed a non-contact infrared temperature measurement system and applied it to the access control platform in the community. As the distances between pedestrians and detective equipment are fixed, the influence of distance on temperature measurement accuracy is reduced. In order to ensure social safety in public areas, Rosepreet et al. [30] proposed an infrared body temperature detection method based on CNN. Non-contact pedestrian body temperature detection was achieved by this method, and the system was applied to the tunnel scenario, improving the detection accuracy by 21.4%.

In the above research achievements, many pedestrian detection methods rely on the high quality of pedestrian datasets. Meanwhile, the data fusion algorithm will be limited by the fixed scenarios. Crowd density estimation is still mainly based on the bird’s-eye-view data, which are obtained by surveillance cameras. However, automated-vehicle-based estimation methods are still to be researched. Additionally, there are difficulties in detecting body temperature at the unrestricted distance, and environmental temperature has a large impact on the accuracy of body temperature detection.

Therefore, we propose a novel method to solve the above problems, and the main contributions in pedestrian detection, crowd density estimation and body temperature detection are summarized as follows:

1. A multi-sensor data fusion method is proposed to improve the accuracy of large, medium and small pedestrian target detection, which is suitable for more scenarios.
2. Based on two-dimensional (2D) foreground image data and three-dimensional (3D) point cloud data, a multi-layer fusion method is designed for crowd density estimation, which can improve the accuracy and intuitiveness of estimation.
3. Based on the foreground image, crowd targets are detected to fix the distance between the sensor and the crowd target. Furthermore, an empirical method, external bold body method and outlier filtering method are proposed to correct the temperature detection results.

## 3. Pedestrian State Sensing Method

**Figure 1:**
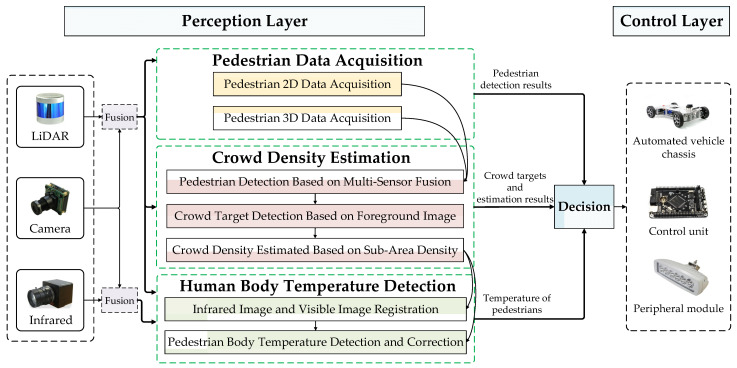
Structure of advanced pedestrian state sensing method for automated patrol vehicle based on multi-sensor fusion.

Based on intelligent automated vehicles and information fusion technology, an advanced pedestrian state sensing method for automated patrol vehicle based on multi-sensor fusion is proposed to reduce the risk of cross-infection in the epidemic prevention and control areas. Pedestrian state includes pedestrian target, crowd aggregation and pedestrian body temperature. Hence, our method is designed in three parts, including pedestrian data acquisition, crowd density estimation and pedestrian body temperature detection. The method includes sensing equipment, decision equipment and control equipment. LiDAR, camera and infrared are mounted on the body of the automated patrol vehicle as the sensing devices; the industrial personal computer (IPC) with the robot operating system (ROS) is used as the decision-making device; the STM32 Microcontroller is used as the control device. The detection of pedestrian and crowd objects, the estimation of crowd density and the detection of pedestrian body temperature are achieved through the detection method proposed in this paper. The structure of proposed method is shown in Figure 1.

### 3.1. Pedestrian Data Acquisition

How to acquire the pedestrian data is the basis of the proposed method in this paper, which consists of two parts: (1) 2D pedestrian data acquisition based on YOLO V4 algorithm; (2) 3D pedestrian data acquisition based on Euclidean clustering algorithm. The obtained data can be used as the data support for crowd density estimation and temperature detection algorithm.

#### 3.1.1. Pedestrian 2D Data Acquisition

**Figure 2:**
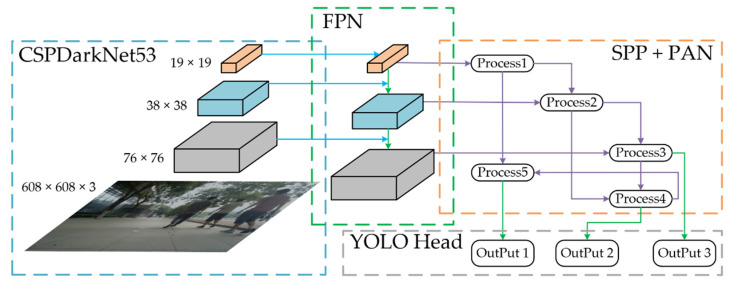
Structure of pedestrian detection based on YOLO V4.

The 2D pedestrian data include pedestrian detection box and face detection box based on visual algorithm. To implement the pedestrian detection with strong timeliness and high precision in a specific enclosed area, the Darknet framework based on the YOLO algorithm is used to obtain 2D image data of pedestrians with different occlusion. Since the spatial pyramid pooling (SPP) is adopted in YOLO V4, the network and loss function are optimized, and the intersection over union (IoU) [31] can reflect the degree of intersection between two boxes accurately. Therefore, YOLO V4 is selected as the visual model of proposed pedestrian detection and face detection algorithm. The structure of pedestrian detection based on YOLO V4 is shown in Figure 2.

In order to obtain 2D pedestrian detection box, the pedestrian dataset of the Massachusetts Institute of Technology (MIT) and the data of pedestrians in the campus environment collected by us are adopted as the input dataset of YOLO network training. Through adjusting the brightness and saturation of the pedestrian image, the learning ability and environmental resistance of pedestrian detection model can be improved. In order to solve the mesoscale problem of target detection, feature pyramid network (FPN) is used to construct a pyramid on the feature graph. Similarly, to reduce the amount of calculation and ensure the accuracy, a cross-stage partial (CSP) module is added to each Darknet53 in YOLO network, pedestrian features are extracted by the first 52 layers and fusion features are output by the last layer. In order to prevent overfitting, dropblock regularization is used to randomly drop features in the same pedestrian module. The SPP module and path aggregation network (PANet) module are added between the feature extraction network and the final output layer to enhance pedestrian feature level and complete multi-scale fusion. Finally, the large, medium and small pedestrian targets are predicted, and pedestrian detection boxes are obtained.

**Figure 3:**
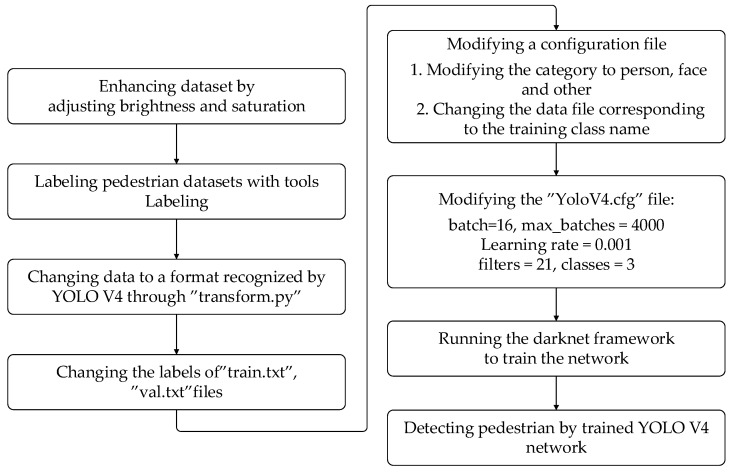
Flow chart of training YOLO V4 network and face detection.

In order to obtain face detection boxes, it is necessary to train the YOLO V4 network with a dataset containing facial features. The MIT pedestrian dataset does not contain facial feature information. Therefore, the dataset with face features needs to be added. As Celeba (an open-source dataset of pedestrian faces) [32] does not take up too many resources and the accuracy of face detection box acquisition satisfies the requirements of this paper, Celeba is selected to add to the dataset. The flow chart of training YOLO V4 network and achieving face detection is shown in Figure 3. During training, the adjusted brightness and saturation can improve the robustness of the algorithm. Firstly, we modify Celeba to a dataset format, which can be recognized by YOLO V4. Then, the configuration files, such as “transform.py”, “train.txt” and “val.txt”, are modified. The number of classifications is adjusted to three types, and the learning rate is adjusted to 0.001. Finally, face detection boxes can be obtained through the YOLO V4 network trained by Celeba.

#### 3.1.2. Pedestrian 3D Data Acquisition

**Figure 4:**
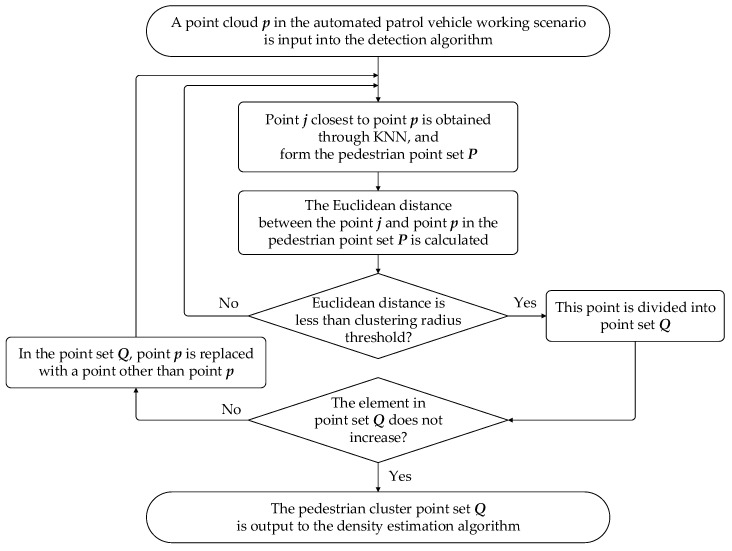
Flow chart of pedestrian point cloud detection based on Euclidean clustering algorithm.

Considering the insufficient accuracy of the YOLO V4 algorithm for long-distance detection, LiDAR is used to obtain the 3D pedestrian data to increase the dimension of data. Euclidean clustering algorithm is applied to obtain 3D pedestrian detection boxes, which contain three primary parameters in this algorithm. It includes the threshold of clustering radius R, the minimum number of clustering points $P_{\min}$ and the maximum number of clustering points $P_{\max}$. The flow chart of pedestrian point cloud detection based on Euclidean clustering algorithm is shown in Figure 4.

In order to obtain 3D pedestrian data through Euclidean clustering, the threshold needs to be set first. Considering the sparse characteristic of cloud point data, different thresholds of clustering radius R are set according to the sparsity of point cloud at different distances. Details of the relationship between pedestrian distance $L_{peds}$ and cluster radius threshold R are listed in Table 1.

Then, the set of points to be searched in the point cloud space is defined as P. According to the features of P, we create a 3D k-dimensional tree (KD-Tree) object. A feature point $P_{i}={\left(x_{i},y_{i},z_{i}\right)}^{T}$ is selected from P, its nearest neighbor points $P_{n}=\{P_{j},j=1,2,\ldots ,n\}$ are searched and the Euclidean distance $D_{ij}$ between points $P_{i}$ and $P_{j}$ is calculated through K-nearest neighbor (KNN) algorithm. The Euclidean distance calculation function is represented as Equation (1):

(1) $$D_{ij}=\sqrt{{\left(x_{i}-x_{j}\right)}^{2}+{\left(y_{i}-y_{j}\right)}^{2}+{\left(z_{i}-z_{j}\right)}^{2}}$$

where $x_{i}$, $y_{i}$ and $z_{i}$ indicate the coordinates of the *i*-th point; $x_{j}$, $y_{j}$ and $z_{j}$ indicate the coordinates of the *j*-th point. Once the Euclidean distance $D_{ij}$ is less than the threshold of clustering radius R, its corresponding point $P_{j}$ is classified as a clustered point cloud set Q. According to the relationship between its Euclidean distance and the threshold, determine whether the point belongs to the clustered point cloud set Q.

Finally, according to the preset minimum number of clustering points $P_{\min}$ and maximum number of clustering points $P_{\max}$, we judge whether the clustered point cloud set Q can be used as a valid single pedestrian target. If the number of point clouds in Q is greater than $P_{\min}$ and less than $P_{\max}$, the cluster point cloud set Q is valid. Meanwhile, a pedestrian clustering result is generated and the 3D pedestrian data are obtained.

### 3.2. Crowd Density Estimation

At present, the crowd aggregation is an important factor causing cross-infection. Crowd aggregation is related to the crowd density of a scenario. In order to locate crowded scenarios accurately, the 2D and 3D pedestrian data obtained in the previous section are combined to improve the accuracy of the estimation algorithm. A multi-layer fusion method is designed for crowd density estimation, which consists of three parts: (1) pedestrian detection based on multi-sensor fusion, (2) crowd target detection based on foreground image and (3) crowd density estimation based on sub-area density.

#### 3.2.1. Pedestrian Detection Based on Multi-Sensor Fusion

**Figure 5:**
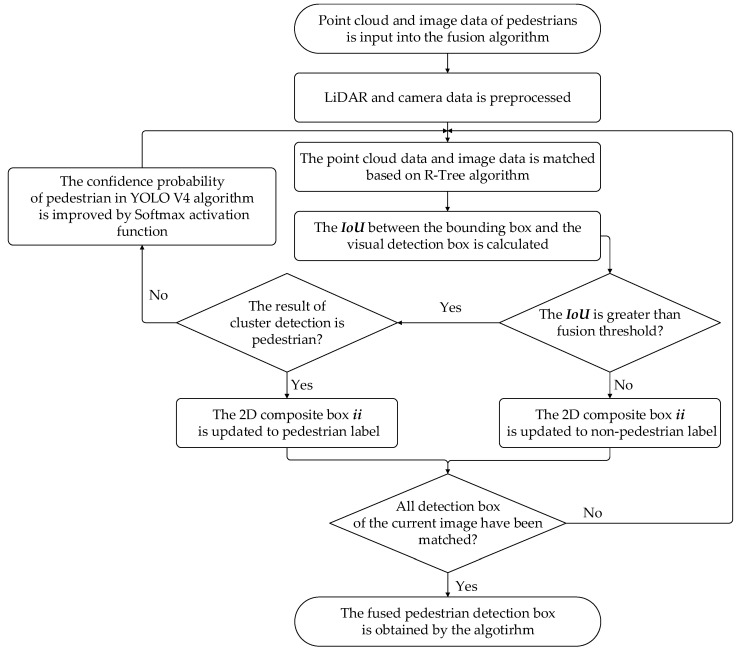
Flow chart of multi-sensor data fusion based on confidence optimization.

After acquiring the 2D and 3D pedestrian data, multi-source data of pedestrians are fused based on the confidence optimization. The confidence of the pedestrians, applied in the YOLO V4 algorithm, has a great influence on the algorithm’s detection results for pedestrians. The value of confidence is related to the constraints of pedestrian features in the YOLO V4 algorithm. The increase in confidence indicates that the constraints of the current pedestrian are more stringent, which will reduce FP rate. However, due to strict constraints, pedestrians with no obvious features may be missed by the algorithm. Therefore, in order to achieve accurate detection of long-distance and occluded pedestrians, the pedestrian confidence probability of the YOLO V4 algorithm is corrected by the Euclidean clustering algorithm. The flow chart of multi-sensor data fusion based on confidence optimization is shown in Figure 5.

Firstly, the preprocessing of the fusion algorithm is achieved based on Zhang calibration [33] method and timestamp matching; secondly, the point cloud with the pixel data is matched through the R-Tree algorithm and the IoU between the bounding box and the 2D image detection box is calculated to determine the numerical relationship between the value of the IoU and the threshold. Finally, through the activation function Softmax(), the confidence probability of pedestrians in the image detection algorithm is corrected, which realizes the fusion of multi-sensor data. The fusion results can be used as a data source for crowd density estimation.

(1) Multi-sensor data preprocessing

**Figure 6:**
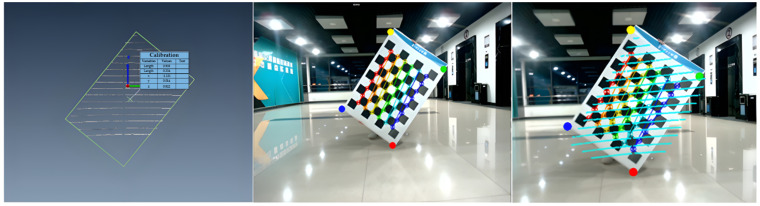
The coordinate system calibration of the multi-source sensors.

The 2D and 3D detection boxes are input into the preprocessing part once the fusion algorithm is starting. Through Zhang calibration method, we obtain the relationship between the pixel coordinate system and the point cloud coordinate system. The coordinate system calibration of the multi-source sensors is shown in Figure 6. The four dots in different colors represent the vertices of the calibration board, and the lines in different colors represent the internal parameters of the camera and the degree of distortion. Through the calculation of the rotation vector and the translation vector, the matching of the image and the point cloud is achieved. The matching of timestamp is resolved by the Time Synchronizer mechanism. Since the point cloud sampling rate is 10 Hz, the image sampling rate is 25 Hz. Therefore, we add timestamps to each frame of data while collecting data from LiDAR and camera and call back the data with the same timestamp.

(2) Point cloud data and pixel data matching

The R-Tree algorithm is used to associate and match the detection box of the point cloud and the image to project the point cloud data to the pixel plane. According to the IoU of the image detection result and the projection result, the specific category information of the detected target in the point cloud space can be determined since there are several detection boxes, respectively, in each frame of image and point cloud data. We select a separate frame for matching each time, and the steps of the fusion algorithm are as follows:

Step 1: A frame of image data and point cloud data, including m 3D detection boxes and n 2D detection boxes, are input into the algorithm;

Step 2: The *i*-th 3D detection box and the *j*-th 2D detection box are selected. We set the initial values of parameters i and j to 1;

Step 3: The points in the *i*-th 3D detection box are projected onto the image to generate a 2D bounding box ii;

Step 4: The IoU of the 2D bounding box ii and the *j*-th 2D detection box are calculated through the algorithm. Through comparing the value of IoU of the two boxes and the threshold, we can determine the category information of the detection result.

IoU value is calculated as shown in Equations (2)–(4):

(2) $$intersection\;=\left(\min\left(x_{ii}^{a_{2}},x_{j}^{b_{2}}\right)-\max\left(x_{ii}^{a_{1}},x_{j}^{b_{1}}\right)\right)\times \left(\min\left(y_{ii}^{a_{2}},y_{j}^{b_{2}}\right)-\max\left(y_{ii}^{a_{1}},y_{j}^{b_{1}}\right)\right)$$

(3) $$union=S_{ii}+S_{j}-intersetion$$

(4) $$IoU=\frac{intersection\;}{union}$$

where intersection indicates the intersection between the bounding box and the 2D detection box, $\left(x_{ii}^{a_{1}},y_{ii}^{a_{1}}\right)$ indicates the coordinates of the upper left corner of the bounding box, $\left(x_{ii}^{a_{2}},y_{ii}^{a_{2}}\right)$ indicates the coordinates of the lower right corner, $\left(x_{j}^{b_{1}},y_{j}^{b_{1}}\right)$ indicates the coordinates of the upper left corner of the 2D detection box, $\left(x_{j}^{b_{2}},y_{j}^{b_{2}}\right)$ indicates the coordinates of the lower right corner, union indicates the union between two 2D detection boxes, $S_{ii}$ indicates the area of the point cloud detection box after mapping and $S_{j}$ indicates the area of the 2D detection box.

(3) Pedestrian confidence correction

The fusion threshold of the intersection ratio is set to 0.75 [34], and the value of the IoU is compared with the fusion threshold to judge whether the current bounding box represents the pedestrian target. If the IoU is less than the fusion threshold, the label of the 2D bounding box is updated to be non-pedestrian; if the current IoU is greater than the fusion threshold and the clustering detection result is non-pedestrian, the confidence probability of the target is increased based on activation function Softmax(). Through adjusting the pixel and point cloud weight values, the correction effect is achieved. The confidence probability correction function is represented as Equation (5):

(5) $$P=\max(Softmax(W_{LiDAR},W_{vision})=\frac{e^{W_{LiDAR}}}{\sum_{i=1}^{n}e^{W_{i}}})$$

where P indicates the value of probability correction, $W_{i}$ indicates the value of i-th sensor weight, where i includes vision and LiDAR, and n indicates the number of sensors.

#### 3.2.2. Crowd Target Detection Based on Foreground Image

**Figure 7:**
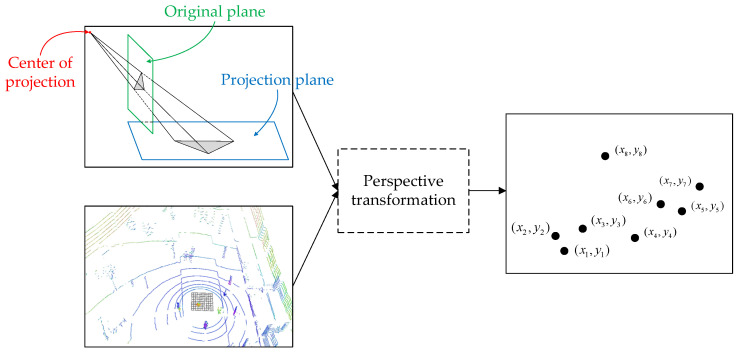
Structure of perspective transformation.

Crowd aggregation is one of the sources of virus spread, and current body temperature detection without distance restriction is difficult. Therefore, it is necessary to detect the aggregated crowd first and then detect the temperature of pedestrians in real time. Camera mounted on automated patrol vehicle can only acquire foreground image data. Due to occlusion and lack of depth information, the accuracy of detecting crowd targets based on foreground images cannot satisfy the patrol requirements. The 3D point cloud data has advantages in spatial sensing of the environment. Since the crowd target consists of multiple single pedestrians, we select the Euclidean clustering algorithm to detect the crowd target. Therefore, based on LiDAR and camera, perspective transformation algorithm is designed. The structure of perspective transformation is presented in Figure 7.

Through perspective transformation, the pedestrian detection box in the 2D image is mapped to a point in the top view. The perspective transformation function is represented as Equation (6):

(6) $${\left[\begin{array}{l} Y_{i}\\ 1 \end{array}\right]}_{(i+1)\times 1}=\left[\begin{array}{l} \begin{array}{cc} A_{2\times 2} & t_{2\times 1} \end{array}\\ \begin{array}{cc} 0_{1\times 2} & E_{1\times 1} \end{array} \end{array}\right]{\left[\begin{array}{l} X_{i}\\ 1 \end{array}\right]}_{(i+1)\times i}$$

where $Y_{i}$ takes the corner coordinate values of the three groups of detection boxes after transformation, $X_{i}$ takes the corner coordinate values of the three groups of detection boxes before the transformation and A represents the matrix of the transformation.

Combined with the point cloud data, we assign the coordinate information of each target to the mapped pedestrian target points. The point set is clustered by the Euclidean clustering algorithm. The KNN algorithm based on KD-Tree object is an important preprocessing method of Euclidean clustering algorithm. Due to the fact that the errors in the parameter estimation process can be avoided by this algorithm and this algorithm has high classification accuracy, KD-Tree is used to traverse the adjacent pedestrian targets and obtain the minimum distance between pedestrians. The algorithm flow of the minimum distance calculation between pedestrians in 3D space is shown as follows:

Step 1: The pedestrian target (3D target recognition box, confidence degree) obtained by the data fusion algorithm is used as the input of the algorithm;

Step 2: The centroid coordinates of each pedestrian target $\left(X_{Peds},Y_{Peds},Z_{Peds}\right)$ are extracted by the algorithm, and the dimension of the current pedestrian centroid data n is calculated as the set;

Step 3: The dimension d with the largest variance and the median m of all data items on dimension d are found. The dataset is divided according to the median m, which is divided into two parts, and the two data subsets are recorded as $D_{l}$ and $D_{r}$, respectively;

Step 4: A pedestrian centroid KD-Tree node is established to store the division dimension d and the median m. Step 2 is performed again on the data subsets $D_{l}$ and $D_{r}$ divided by the Step 3, and the newly generated centroid KD-Tree node is set as the left and right child nodes;

Step 5: The dataset is continuously divided until the pedestrian centroid data subset contains less than two pieces of data after the current division. The corresponding centroid data are saved to the last leaf node, which is the KD-Tree object of the pedestrian centroid;

Step 6: Each pedestrian target is traversed by the KNN algorithm to find the current pedestrian and the nearest pedestrian. The Euclidean distance between the current pedestrian and the closest pedestrian is calculated through KNN algorithm;

Step 7: Pedestrian minimum distance sequence is obtained by minimum distance calculation algorithm.

**Figure 8:**
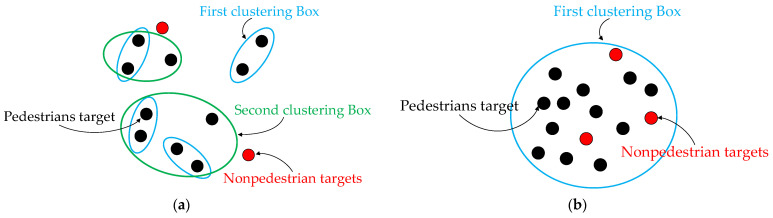
Detection results of crowd target. (**a**) Euclidean cluster in Condition 1; (**b**) Euclidean cluster in Condition 2.

The clustering threshold is one of the most important parameters in Euclidean clustering algorithm, and it is necessary to be set reasonably. Combined with the minimum distance sequence of pedestrians, which are obtained by the above algorithm, the distance features of pedestrians are distinguished. Due to the change in the objective of clustering, the thresholds are set according to the following two clustering conditions existing in the work scenario of the automated patrol vehicle;

Condition 1: There is a long distance between the center point and each clustering point, as shown in Figure 8a;

Condition 2: The center of the cluster point is in the scenario with high crowd density, as shown in Figure 8b.

For Condition 1, the centroid point cloud of each pedestrian is used by the clustering algorithm to represent the current pedestrian target, and the centroid point cloud of all pedestrians is classified into a new clustering space. Through the experiments in Section 4, the clustering threshold is selected as 0.5 m and the pedestrian centroid point cloud is clustered. The requirements of crowd target detection are satisfied. For Condition 2, areas with high density are regarded as complete crowd targets by the clustering algorithm to achieve crowd detection.

As shown in Figure 8, a single black point in the scenario represents the pedestrian target detected by the algorithm, and the red point represents the non-pedestrian target. Firstly, the non-pedestrian target is eliminated through the algorithm. Then, two adjacent pedestrian targets are clustered based on the KNN algorithm. The clustered target is used as a new individual to cluster again until there is no single pedestrian target in the scenario. Finally, crowd targets are obtained as the algorithm output.

#### 3.2.3. Crowd Density Estimated Based on Sub-Area Density

In order to improve the effect of density estimation, a crowd density estimation algorithm based on sub-area density is proposed to expand the difference in the distribution characteristics of crowd density. The layer is divided into equal-area units by the algorithm, and the density values of the units are selected as input of the algorithm. Through fusing crowd density data from multiple layers, the crowd density distribution is calculated. Based on the weighted mean theory, the crowd density of the current scenario is estimated.

According to the number of pedestrian targets detected by the automated patrol vehicle in the current scenario, the layer with a side length of 15 m is divided into 32 × 32 units by algorithm. Each of these units covers an area of 0.22 square meters, which fits the minimum footprint of an adult. The density value of unit is taken from the ratio of the number of pedestrians in each unit to its area of unit, whose calculation is shown as Equation (7):

(7) $$\left\{\rho_{\left(i,j\right)}|\rho_{\left(i,j\right)}=\frac{N_{ij}}{S},i,j\in 50\right\}$$

where $\rho_{\left(i,j\right)}$ indicates the density value of unit, $N_{ij}$ indicates the number of pedestrians in the unit of the *i*-th row and the *j*-th column and S indicates the area of a single unit within the layer. The calculation algorithm of $N_{ij}$ is illustrated in Algorithm 1.
**Algorithm 1:**$N_{ij}$ calculation algorithm in 3D space.1:Initialize pedestrian detection results, target detection boxes and confidence level.2:Create a List $L_{\mathrm{targets}}$ of every unit, a centroid variable *C* and a pedestrian count variable $N_{count}$.3:**While** ($i_{\min}$ < i < $i_{\max}$ && $j_{\min}$ < j < $j_{\max}$) **do**4:  **If (**target detection boxes**) then**5:    $L_{\mathrm{targets}}$ = $L_{\mathrm{targets}}$ + 16:  **for each**
$L_{\mathrm{targets}}$ **do**7:    **If** (*C* is under the range of the corner coordinates of $L_{\mathrm{targets}}$) **then**8:      $N_{count}$
$=N_{count}$ + 19:    **end if**
10:  **end for**
11:  $N_{ij}$ =$N_{count}$
12:**End while**13:Return $N_{ij}$


The initial crowd density distribution matrix $M_{\mathrm{old}}$ is composed of the density values of unit $\rho_{\left(i,j\right)}$. The numerical values in each row and column of the matrix are the density values of unit. The initial crowd density distribution matrix is represented as Equation (8):

(8) $$M_{old}=\left[\begin{array}{cccc} \rho (1,1) & \rho (1,2) & \ldots & \rho (1,j)\\ \rho (2,1) & \ddots & \rho (2,j-1) & \rho (2,j)\\ \vdots & \rho (i-1,2) & \ddots & \rho (i-1,j)\\ \rho (i,1) & \rho (i,2) & \ldots & \rho (i,j) \end{array}\right],i,j\in 50$$

**Figure 9:**
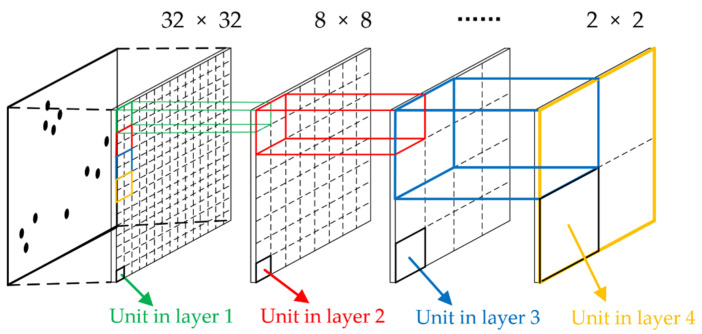
Structure of crowd density distribution detection based on the multi-layer fusion algorithm.

The initial crowd density distribution matrix is represented by the current density of each individual point. However, density is one of the attributes of the crowd; the arrangement of density values of individual points is not representative. Therefore, the density features of adjacent units in the layer are correlated by this algorithm. After the corresponding unit density values of different layers are linearly combined, the unit density values containing the features of adjacent units are obtained. The matrix $M_{new}$, which is composed of each unit density value containing adjacent unit features in the layer, is the final crowd density distribution matrix. The structure of crowd density distribution detection based on the multi-layer fusion algorithm is shown in Figure 9.

A 2D coordinate system is established according to the initial position of the automated patrol vehicle in Section 3.2.1. Along the initial direction of the automated patrol vehicle, the 2 × 2 units are sequentially taken to form a new unit in the next layer, a new layer of 16 × 16. The side length of any 2 × 2 unit in the current layer is 0.9 m. The pedestrian density value in the 2 × 2 unit is counted and superimposed with the density value of the unit in upper layer. The superposition algorithm is represented as Equation (9):

(9) $$\rho_{\left(i,j\right)}^{k}=\sum_{i=1,j=1}^{2}\rho_{\left(i,j\right)}^{k-1}$$

where $\rho_{\left(i,j\right)}^{k}$ indicates the density value of the unit in the *i*-th row and the *j*-th column after the *k*-th division, and $\rho_{\left(i,j\right)}^{k-1}$ indicates the density value of the unit of the *i*-th row and the *j*-th column after the (*k* − 1)-th division.

It is worth mentioning that the total area of the single layer does not change after the layer is divided by the above algorithm. The 2 × 2 units are finally iterated by the algorithm. We count the density values of the units after each division, and, based on the fusion of multi-layer by weighting, the density distribution matrix $M_{new}$ is obtained as the output of the algorithm. The weighted superposition is represented as Equation (10):

(10) $$M_{new}=\left[\begin{array}{cccc} \sum_{k=0}^{n}w_{k}\cdot \rho_{\left(1,1\right)}^{k} & \sum_{k=0}^{n}w_{k}\cdot \rho_{\left(1,2\right)}^{k} & \ldots & \sum_{k=0}^{n}w_{k}\cdot \rho_{\left(1,j\right)}^{k}\\ \sum_{k=0}^{n}w_{k}\cdot \rho_{\left(2,1\right)}^{k} & \ddots & \sum_{k=0}^{n}w_{k}\cdot \rho_{\left(2,j-1\right)}^{k} & \sum_{k=0}^{n}w_{k}\cdot \rho_{\left(2,j\right)}^{k}\\ \vdots & \sum_{k=0}^{n}w_{k}\cdot \rho_{\left(i-1,2\right)}^{k} & \ddots & \sum_{k=0}^{n}w_{k}\cdot \rho_{\left(i-1,j\right)}^{k}\\ \sum_{k=0}^{n}w_{k}\cdot \rho_{\left(i,1\right)}^{k} & \sum_{k=0}^{n}w_{k}\cdot \rho_{\left(i,2\right)}^{k} & \ldots & \sum_{k=0}^{n}w_{k}\cdot \rho_{\left(i,j\right)}^{k} \end{array}\right],i,j\in 50$$

where $w_{k}$ indicates weight of the *k*-th division. In order to improve the detection efficiency, the harmonic series is used as the weight update function to magnify the difference between the crowd density in different units. The weight update function is represented as Equation (11):

(11) $$w_{k}=\frac{1}{k+1},k\in [0,N]$$

where k indicates the current superposition times and N indicates the total number of divisions.

Real-time detection in disease control areas is very important. Due to the multi-dimensional nature of crowd features, the weighted mean theory used in the algorithm should have high real-time performance and the density estimation should satisfy the requirements of patrolling. Therefore, we combine the comparative requirements of the crowd density of different layers, and, based on the weighted mean theory, estimate the crowd density in the current scenario. The estimation equation is represented as Equation (12):

(12) $$\rho_{crowd}=\frac{\sum_{i}\sum_{j}\sum_{k=0}^{n}w_{k}\cdot \rho_{\left(i,j\right)}^{k}}{\iint d\sigma },i,j\in 50$$

where $\rho_{crowd}$ indicates the crowd density and σ indicates the overall area of the layer.

### 3.3. Pedestrian Body Temperature Detection

**Figure 10:**
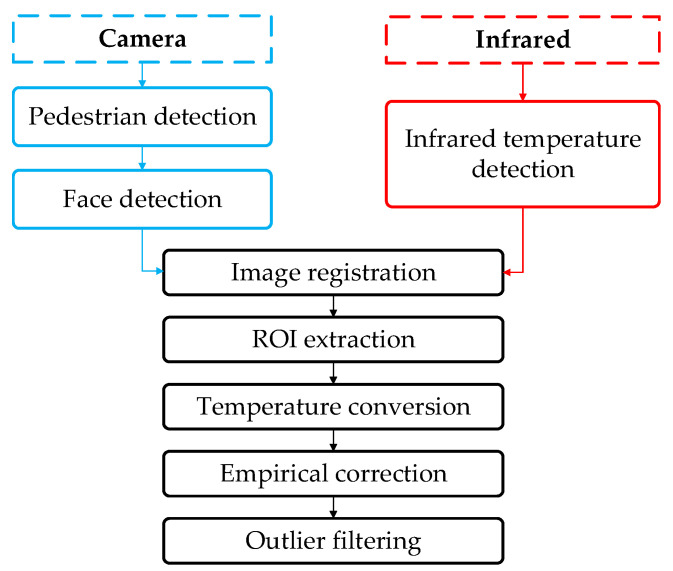
Flow chart of pedestrian body temperature detection.

At present, more than 40% of the patients that test positive for the coronavirus have high temperature features in their clinical manifestations [35], and the risk of current epidemic spreading can be decided by temperature. Therefore, a body temperature detection algorithm based on thermal imaging is designed after the density estimation to ensure the risk of cross-infection can be reduced by temperature detection with high crowd density. Since the accuracy limitations of current temperature sensors, it is difficult to detect temperature at any distance accurately. Therefore, in order to reduce the cost of temperature detection and improve the accuracy, the distance from the sensor to the target to be detected is fixed at 3 m, and an external bold body, an empirical method and outlier filtering are used to correct the detection results. The flow chart of pedestrian body temperature detection is shown in Figure 10.

#### 3.3.1. Infrared Image and Visible Image Registration

In order to combine image data after fusion and infrared data, the registration method is used to fuse multi-sensor data. Color image data is output by the infrared camera in real time, in which each pixel represents the temperature value. Through detecting the temperature value of the face area, we calculate the current pedestrian’s body temperature. However, since the angle and orientation of the output images of infrared and visible images to the same target are different, it is necessary to register the infrared image and the visible image. Several matching points in the infrared and visible images are selected, and we register the two images by means of affine transformation. Affine transformation function is shown in Equation (13):

(13) $$\left[\begin{array}{l} {x^{\prime }}_{i}\\ y_{i}{}^{\prime } \end{array}\right]=A\left[\begin{array}{l} x_{i}\\ y_{i} \end{array}\right]+BI$$

where A and B indicates the parameters of affine transformation equation, and $\left(x_{i},y_{i}\right)$ and $\left(x_{i}{}^{\prime },y_{i}{}^{\prime }\right)$ indicate a set of corresponding coordinates in visible and infrared images. The equation of obtaining parameters A and B of affine transformation equation is shown in Equation (14):

(14) $$\left\{ \begin{array}{l} A=\left[\begin{array}{cc} \begin{array}{c} a_{11}\\ a_{21} \end{array} & \begin{array}{c} a_{12}\\ a_{22} \end{array} \end{array}\right]\\ B=\left[\begin{array}{l} b_{1}\\ b_{2} \end{array}\right] \end{array}\right.$$

In order to determine the two parameters in the process of image registration in the above equation, different corners of the detection screen are obtained by the automated patrol vehicle in real time. When the number of extracted corners is more than three groups, the optimal parameters of affine transformation equation can be fitted by the least square method or gradient descent method. Since the least square method can be used to find the global solution instead of the local optimal solution in the gradient descent method. Therefore, multiple matched corners are selected to fitting the optimal solution based on the least square method. Infrared image and visible image are registered by the equation after obtaining the parameters of affine transformation equation.

#### 3.3.2. Human Body Temperature Detection and Correction

The corresponding point can be found both in the infrared image and the visible image after the registration, which can establish the mapping relationship to match the face area. Considering the accuracy of the infrared sensor, we set the distance of the temperature detection between 3–8 m. However, there are temperature interference factors such as hats, glasses and hair in the actual scenario, which leads to its temperature lower than the real value. Therefore, through the 5 × 5 template in the region of interest (ROI), the mode index of temperature of each template in the current scenario is calculated by the algorithm. The template is averaged as the pedestrian’s body temperature. The preset template is used to traverse the face area after the face target is detected in the dense area. And temperature data are extracted for each pixel within the template. The temperature of pedestrian calculation method is represented as Equation (15):

(15) $$T_{i}=\frac{\sum_{j=1}^{n}M(j)}{n}$$

where $T_{i}$ indicates the temperature of *i*-th pedestrian, n indicates the number of face detection box pixels and M(j) indicates the mode of infrared detection temperature in a single 5 × 5 template.

Meanwhile, hardware errors are still inevitable, and the algorithm detection results are quite different from the values detected by the thermometer. Considering that the most accurate body temperature detection is the thermometer, it is reasonable to use it as the standard. Therefore, we combined the algorithm detection results with the thermometer detection results to correct the body temperature detection algorithm by empirical method. The temperature deviation caused by the occlusion of glasses and other facial decoration can be reduced effectively. And the local abnormal temperature caused by the sensor distance accuracy can be reduced by taking the mean value. The correction algorithm is shown as Equation (16):

(16) $$T_{p}=T_{f\_d}+\sqrt{\frac{T_{f\_d}^{2}}{T_{f\_d}^{2}+{\left(T_{f\_d}-T_{env}\right)}^{2}}}\times (T_{t}-\frac{\sum_{i=1}^{n}T_{i}}{n})$$

where $T_{p}$ indicates the detection temperature obtained after correction, $T_{f\_d}$ indicates the result of infrared temperature measurement, $T_{env}$ indicates the current environmental temperature, $T_{t}$ indicates the current pedestrian’s body temperature measured by a thermometer and n indicates the number of selected measurements.

In addition, errors in the temperature detection are mainly due to temperature drift caused by environmental changes and long-term operation. The probe of an infrared detector is exposed in the infrared radiation, including air temperature and radiation generated by the surrounding environment. Due to changes in the environment, the state of infrared radiation is changed, which will reduce the accuracy of temperature detection. The long-term work of automated patrol vehicles can also cause temperature drift. Therefore, we adopt external bold body correction and outlier filtering to further improve the accuracy of temperature detection. By setting the external bold body in front of the infrared camera, the error of temperature detection can be reduced and the stability of the system can be improved. The external bold body correction coefficient function is represented as Equation (17):

(17) $$K=\frac{T_{0}}{T_{a}}$$

where K indicates the temperature correction coefficient, $T_{0}$ indicates the absolute temperature set by the external bold body and $T_{a}$ indicates the absolute temperature detected by the external bold body. The external bold body is a constant temperature source, whose temperature $T_{0}$ is fixed at 37°.

Moreover, outliers are usually caused by pedestrian occlusion, face detection errors, environmental temperature changes and other factors in the process of body temperature detection. In order to ensure the accuracy of temperature detection, temperature detection values of less than 35° and greater than 42° are removed with reference to the normal body temperature of pedestrians.

## 4. Experimental Results and Discussion

**Figure 11:**
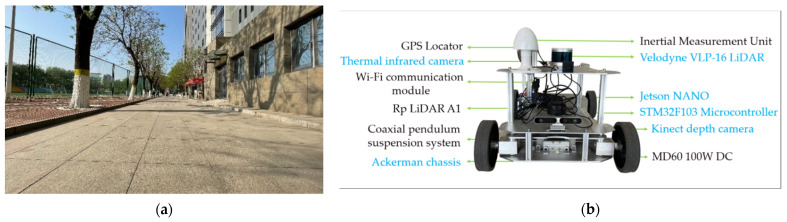
Experimental platform based on automated patrol vehicle. (**a**) Experimental scenario. (**b**) Automated patrol vehicle.

In this section, a detection platform and scenario are constructed based on the automated patrol vehicle. Considering the current situation of the pandemic, a straight road in the campus is chosen for the experiment. There are entrances for pedestrians on the north and south sides of the straight road. There are convenience stores and other factors at the end of the east side that cause pedestrian aggregation, as shown in Figure 11a. For this scenario, we design an automated patrol vehicle applied with a detection method. The automated patrol vehicle is equipped with a mechanical 16-line LiDAR, a 720P USB industrial camera, a thermal infrared camera, an Ackerman vehicle chassis, an industrial computer and a monitor screen, as shown in Figure 11b. The measuring range of the mechanical LiDAR does not exceed 150 m, and the minimum measuring range is 0.5 m. It is mounted perpendicular to the vehicle at 0.5 m. The camera is mounted perpendicular to the LiDAR at 0.4 m directly below. The thermal infrared camera is mounted perpendicular to the camera at 0.6 m directly below. The maximum velocity of the automated patrol vehicle is 4 m per second, which satisfies the security requirements of patrolling on campus.

Then, the feasibility and accuracy of pedestrian data acquisition, crowd density estimation and real-time body temperature detection are analyzed and discussed.

**Figure 12:**
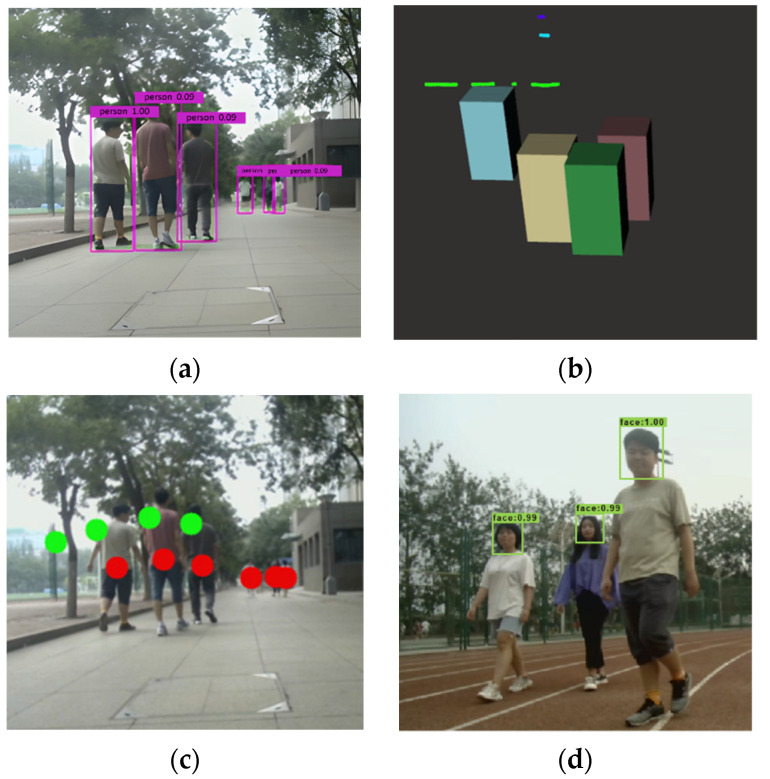
The results of pedestrian detection and face detection. (**a**) 2D detction box. (**b**) 3D detection box. (**c**) Fusion results. (**d**) Face detection results.

The experiments of pedestrian data acquisition consist of two parts: (1) feasibility of pedestrian data acquisition; (2) pedestrian detection accuracy verification. The 2D pedestrian data acquisition results using the YOLO V4 algorithm are shown in Figure 12a, and the rectangular boxes are the detection results of pedestrians. The 3D pedestrian data acquisition results using the European clustering algorithm are shown in Figure 12b, and the different colored stereoscopic boxes are the detection results of pedestrians. The image data after data fusion are shown in Figure 12c. The red dots represent the pedestrian detection results based on YOLO V4, and the green dots represent the pedestrian detection results based on the Euclidean clustering algorithm. The results of face detection based on the YOLO V4 network trained by Celeba are shown in Figure 12d.

As shown in Figure 12, occluded pedestrians can be detected by the trained YOLO V4 network better. Long-distance pedestrians can be detected by the clustering algorithm, and the location information of pedestrians can be obtained. Combined with the multi-sensor features, the detection range and feasibility of the fusion algorithm are higher than those of a single sensor. As shown in Figure 12d, the face targets can be detected by a trained YOLO V4 network accurately.

**Figure 13:**
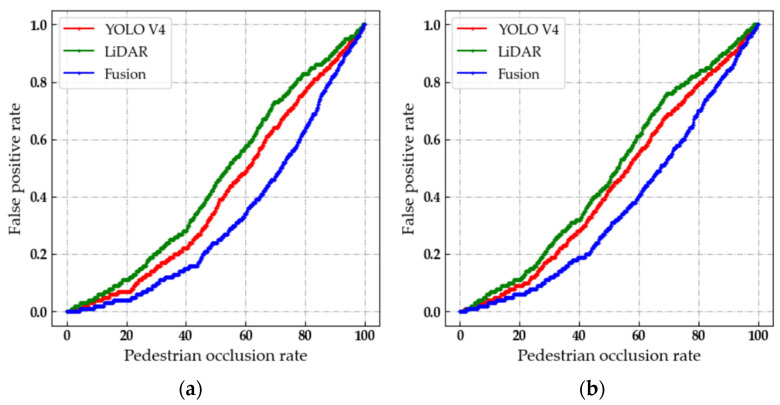
Fitting curves of false positive rate under different scenarios. (**a**) Outdoor environment. (**b**) Academic building.

The FP rate index is used to verify the accuracy of the pedestrian detection algorithm under different pedestrian occlusion rates and different scenarios. Considering the moving velocity of the automated patrol vehicle and the sensing range of the sensor, we fixed the distance between the automated patrol vehicle and the pedestrian target at 10 m. The academic building and outer space are used as the respective experimental scenarios to verify the effects of different lighting and environmental complexity on the detection of pedestrians. In order to verify the detection accuracy after data fusion, the occlusion of pedestrians is defined into four levels [36], including no occlusion for pedestrians, partial occlusion for pedestrians, severe occlusion for pedestrians and complete occlusion for pedestrians. The fitting curves of the false positive rates under different scenarios are shown in Figure 13.

The lower the FP rate of pedestrian targets, the better the detection effect of the fusion algorithm. With the increase in the pedestrian occlusion rate, the FP rate of pedestrian targets will gradually increase. The FP rate of the fusion algorithm is lower than 5% when the pedestrian occlusion rate is below 20% in the academic building and outdoor environment, as shown in Figure 13. Therefore, the fusion algorithm has higher accuracy and stronger robustness compared to the single-sensor detection algorithm. The accuracy of the pedestrian detection method based on the fusion algorithm satisfies the requirements of the crowd density estimation algorithm.

Moreover, we evaluate the effectiveness of crowd density estimation in experimental scenarios through a three-part experiment, which includes: (1) crowd clustering threshold selection and crowd detection implementation; (2) crowd density distribution verification; (3) the accuracy of crowd density estimation verification.

**Figure 14:**
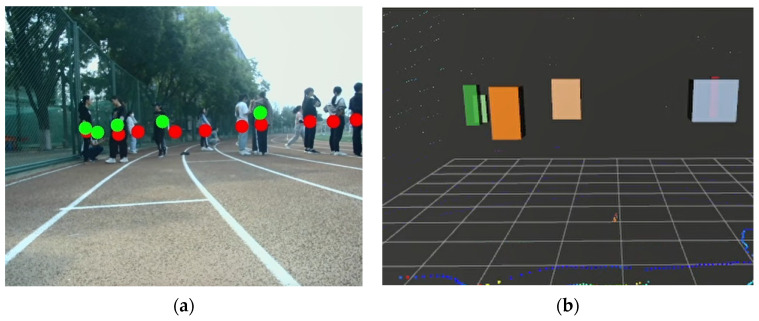
Detection results of crowd target when the clustering threshold is set to 0.5. (**a**) The crowd aggregation state. (**b**) The detection results of crowd target.

The clustering effect of the crowd target is directly related to the clustering threshold. In order to obtain the higher clustering results, a suitable threshold is very important. Therefore, we conduct experiments on the selection of clustering thresholds. The result of the crowd clustering detection with the clustering threshold set to 0.5 is shown in Figure 14. In Figure 14a, the red dots represent the pedestrian detection results based on YOLO V4, and the green dots represent the pedestrian detection results based on the Euclidean clustering algorithm. And in Figure 14b, different colored stereoscopic boxes represent the detection results of crowd targets.

The larger the clustering threshold is, the more blurred the boundary of the crowd target is, as shown in Figure 14. Additionally, the algorithm is more inclined to cluster the scattered points into the detection result, which does not satisfy our requirements. If a small clustering threshold is selected, the number of clustered crowd objects will be very large and will lead to a smaller number of pedestrians contained in a single cluster. The pedestrian targets that cause body temperature to be detected will be reduced, resulting in an increased probability of infection risk. Therefore, we take the clustering threshold as 0.5 m according to the comprehensive judgment of the correlation between the threshold and the number of clusters. The detection results of the crowd target clustering satisfy our requirements under this clustering threshold.

**Figure 15:**
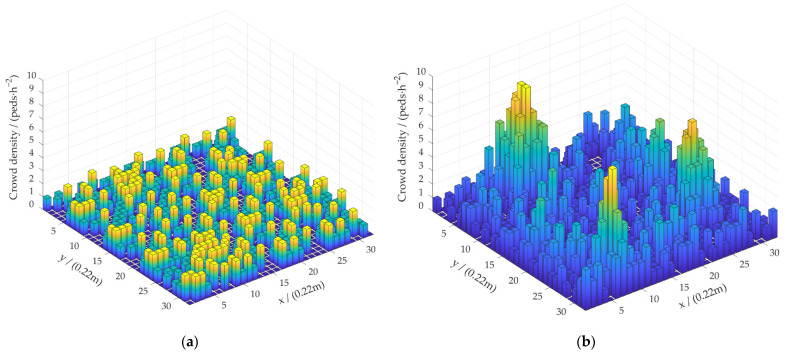
Visualizations of crowd density distribution detection results. (**a**) Initial crowd density distribution $M_{\mathrm{old}}$. (**b**) Final crowd density distribution $M_{\mathrm{new}}$.

The number of pedestrians in the unit will be detected by the automated patrol vehicle. Additionally, combined with the area of unit, the crowd density in the unit will be estimated. We use the 3D thermodynamic chart to show the current distribution of the crowd density in the scenario, as shown in Figure 15. In order to show that our method has better performance, the initial density distribution matrix is used for comparison.

Crowd density is proportional to its thermal peak. Columns with low density values are represented in blue, and the color of the columns transfers to yellow as the density increases. The initial density distribution matrix $M_{\mathrm{old}}$ is shown in Figure 15a, and the final density distribution matrix $M_{\mathrm{new}}$ calculated by our algorithm is shown in Figure 15b. In $M_{\mathrm{old}}$, the aggregation area is not prominent, and the density distribution is uniform. As shown in Figure 15b, there are three peaks in the current scenario. Each peak area represents a group of crowd aggregation. The detection result is consistent with the result of the manual measurement of density distribution. The crowd density in the scenario is estimated based on this distribution. We compare the detection results with the real values measured manually to obtain the density error, as shown in Table 2.

Five crowd groups were preset in the experimental scenario and recorded as five groups of scenarios. The larger the scenario number, the more pedestrians in a single scenario. As the number of pedestrians increases, the value of crowd density increases gradually, as shown in Table 2. The density error is the largest when the scenario number is 4 and the smallest when the scenario number is 1. Since three or more pedestrians are completely occluded in the crowd represented by scenario number 4, the error is the largest. The experimental results have shown that the error of the proposed algorithm in detecting crowd density is 6.54% at maximum and 1.11% at minimum. The detection accuracy satisfies the requirements of crowd density estimation.

**Figure 16:**
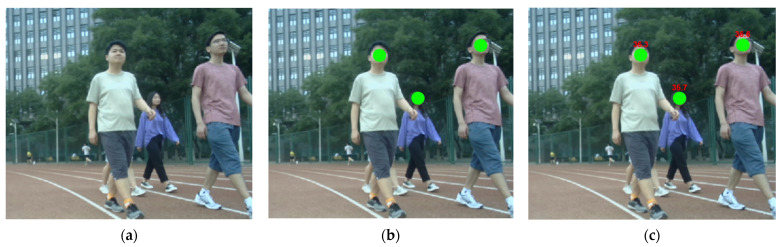
Results of temperature detection. (**a**) Original image. (**b**) Face detection. (**c**) Temperature detection.

The feasibility of the body temperature detection algorithm is verified through two-part experiments, which include: (1) real-time body temperature detection verification; (2) body temperature detection accuracy verification. The body temperature of pedestrians in real time is detected by the automated patrol vehicle. The temperature detection results are displayed visually, as shown in Figure 16.

**Figure 17:**
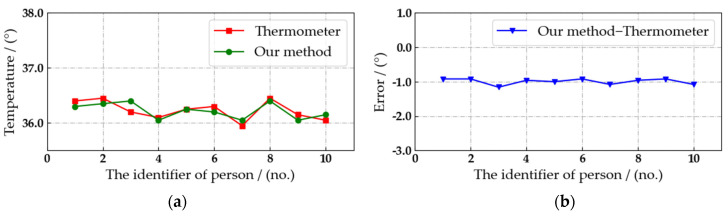
Comparison of pedestrian body temperature detection results at a distance of 3 m. (**a**) Temperature detection results. (**b**) Detection error.

**Figure 18:**
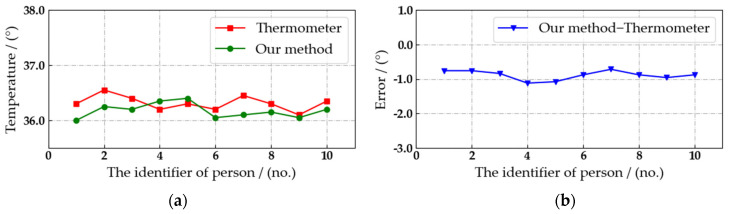
Comparison of pedestrian body temperature detection results at a distance of 4 m. (**a**) Temperature detection results. (**b**) Detection error.

**Figure 19:**
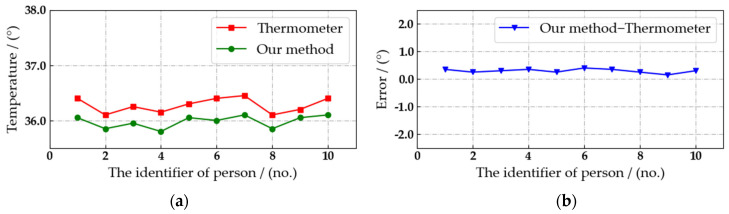
Comparison of pedestrian body temperature detection results at a distance of 5 m. (**a**) Temperature detection results. (**b**) Detection error.

During the experiment, the air temperature is 21.4°. As shown in Figure 16, the real-time body temperature of the current pedestrian can be detected by the automated patrol vehicle, and it can be marked on the pedestrian’s body accurately without abnormal values. Therefore, the proposed algorithm is feasible. Then, the accuracy of our algorithm in detecting pedestrian body temperature is verified through comparative experiments. Considering that the temperature of the same object detected by different devices at different times is not the same, the current results measured by thermometers are highly convincing. Therefore, we select the data measured by the thermometer as the accurate body temperature, and the detection error of the body temperature is calculated through comparing with the detection results, as shown in Figure 17, Figure 18 and Figure 19.

The detection distance is fixed at 3, 4 and 5 m. The interval is set to three seconds, and we select ten consecutive body temperature detection data to compare with the detection results of thermometer. Due to the position of the external bold body and the correction algorithm, the detection of pedestrian body temperature at 3 m is the most accurate, as shown in Figure 17. Comparing the detection results with the body temperature data measured by the thermometer, the absolute value of the error is less than 0.8° in ten records. Considering the safety factors on campus, the automated patrol vehicle has to maintain a reasonable distance from pedestrians. Therefore, the distance between the automated patrol vehicle and the crowd target can be kept at about 3 m, and the body temperature of pedestrians can be detected stably and with high precision at this distance.

The feasibility and accuracy of the pedestrian data acquisition algorithm, the optimal threshold for crowd target clustering, the density estimation and the accuracy of body temperature detection are verified, in turn, after the discussion of the above experiments. The experimental results have shown that the method can work stably in the campus environment.

## 5. Conclusions

In this paper, in order to reduce the risk of cross-infection and improve the accuracy of sensing, we proposed a novel multi-sensor data fusion method, a multi-layer fusion method and a temperature detection method. Meanwhile, the above methods were applied in an automated patrol vehicle, which improves the flexibility of detection. The experimental results demonstrated that the pedestrian detection results are improved by more than 17% compared to the single-sensor algorithm; the density estimation results are more intuitive after applying the multi-layer fusion method. Compared with the thermometer, the error in body temperature detection is less than 0.8°. Due to the limitations of the equipment cost and the volume of the automated patrol vehicle, the detection effect of the proposed method on the lateral and rear targets of the vehicle body needs to be improved, which can be solved by increasing the number of sensors in the future.

## Figures and Tables

**Table 1:** Corresponding relationship between pedestrian distance and clustering radius threshold.

Distance between Pedestrian and the Automated Patrol Vehicle $L_{peds}/(m)$	Threshold of Clustering Radius R/(m)
(0,10)	0.2
(10,20)	0.5
(20,30)	1.0
(30,40)	1.5
(40,50)	2

**Table 2:** Analysis of the accuracy of crowd density estimation.

Scenario Number *n_s_*	The Values of Crowd Density Estimation $\rho_{crowd}/(peds\cdot h^{-2})$	The Error of Crowd Density Estimation $E_{\rho_{crowd}}/(\%)$
1	0.9	1.11
2	2.8	2.52
3	5.3	3.58
4	8.4	6.54
5	10.5	5.91

## Data Availability

All data and models used during the study appear in this article.
